# Association of visceral fat area or BMI with arterial stiffness in ideal cardiovascular health metrics among T2DM patients

**DOI:** 10.1111/1753-0407.13463

**Published:** 2023-09-08

**Authors:** Ling Zhao, Xiangming Zhou, Yufei Chen, Qijuan Dong, Qidong Zheng, Yufan Wang, Li Li, Dong Zhao, Bangqun Ji, Fengmei Xu, Juan Shi, Ying Peng, Yifei Zhang, Yuancheng Dai, Tingyu Ke, Weiqing Wang

**Affiliations:** ^1^ Department of Endocrinology The Second Affiliated Hospital of Kunming Medical University Kunming China; ^2^ Department of Endocrine and Metabolic Diseases Shanghai Institute of Endocrine and Metabolic Diseases, Ruijin Hospital, Shanghai Jiao Tong University School of Medicine Shanghai China; ^3^ Shanghai National Clinical Research Center for metabolic Diseases Key Laboratory for Endocrine and Metabolic Diseases of the National Health Commission of the PR China, Shanghai Key Laboratory for Endocrine Tumor，State Key Laboratory of Medical Genomics, Ruijin Hospital, Shanghai Jiao Tong University School of Medicine Shanghai China; ^4^ Department of Endocrinology People's Hospital of Zhengzhou Affiliated Henan University of Chinese Medicine zhengzhou China; ^5^ Department of Internal Medicine The Second People's Hospital of Yuhuan Yuhuan China; ^6^ Department of Endocrinology and Metabolism Shanghai General Hospital, Shanghai Jiao Tong University School of Medicine Shanghai China; ^7^ Department of Endocrinology Ningbo First Hospital Ningbo China; ^8^ Center for Endocrine Metabolism and Immune Diseases, Beijing Luhe Hospital Capital Medical University Beijing China; ^9^ Department of Endocrinology Xingyi People's Hospital Xingyi China; ^10^ Department of Endocrinology and Metabolism Hebi Coal (group). LTD. General Hospital Hebi China; ^11^ Department of Internal Medicine of Traditional Chinese Medicine Sheyang Diabetes Hospital Yancheng China

**Keywords:** arterial stiffness, ideal cardiovascular health metrics, type 2 diabetes, visceral fat area

## Abstract

**Background:**

“Obesity paradox” occurs in type 2 diabetes mellitus (T2DM) patients when body mass index (BMI) is applied to define obesity. We examined the association of visceral fat area (VFA) as an obesity measurement with arterial stiffness in seven ideal cardiovascular health metrics (ICVHMs).

**Methods:**

A total of 29 048 patients were included in the analysis from June 2017 to April 2021 in 10 sites of National Metabolic Management Centers. ICVHMs were modified from the recommendations of the American Heart Association. Brachial‐ankle pulse wave velocity (BaPWV) ≥ 1400 cm/s was employed to evaluate increased arterial stiffness. Multivariate regression models were used to compare the different effects of BMI and VFA on arterial stiffness.

**Results:**

Lower VFA was more strongly associated with low BaPWV than lower BMI when other ICVHMs were included (adjusted odds ratio [OR], 0.85 [95% confidence interval [CI], 0.80–0.90] vs OR 1.08 [95% CI, 1.00–1.17]). Multivariable‐adjusted ORs for arterial stiffness were highest in patients with the VAT area VFA in the range of 150‐200 cm^2^ (adjusted OR, 1.26 [95% CI 1.12–1.41]). Compared with participants with VAT VFA < 100 cm^2^, among participants with higher VAT VFA, the OR for arterial stiffness decreased gradually from 1.89 (95% CI, 1.73–2.07) in patients who had ≤1 ICVHM to 0.39 (95% CI, 0.25–0.62) in patients who had ≥5 ICVHMs.

**Conclusion:**

In patients with T2DM, using VAT for anthropometric measures of obesity, VFA was more relevant to cardiovascular risk than BMI in the seven ICVHMs. For anthropometric measures of obesity in the ICVHMs to describe cardiovascular risk VFA would be more optimal than BMI.

## Graphical abstract

Highlights

- This is a multicenter study to compare the association of visceral fat area (VFA) or body mass index (BMI) with the presence of arterial stiffness combined with other ideal cardiovascular health metrics (ICVHMs).
- VAT VFA appears to be more relevant to cardiovascular risk and be more optimal than BMI for obesity assessment in the ICVHMs in Chinese type 2 diabetes mellitus patients.

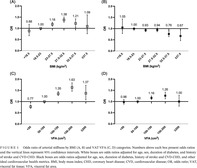

## 1 INTRODUCTION

Cardiovascular disease (CVD) has long been proved to be an important reason of chronic disability and early death worldwide, although mortality due to CVD has declined during the past several decades.^1^ Patients with type 2 diabetes mellitus (T2DM) have approximately a two‐fold increased risk of CVD than individuals without diabetes, with characteristics of earlier occurrence and greater severity.^2^, ^3^ With the dual burden of aging population and COVID‐19, continuous care and management of diabetes are increasing in importance.^4^

In order to improve population cardiovascular health outcomes, the American Heart Association (AHA) therefore recommended a set of seven ideal cardiovascular health metrics (ICVHMs) in 2010. Four healthy lifestyle behaviors (body mass index [BMI], diet, smoking status, and physical activity) and three ideal metabolic measures (fasting blood glucose, blood pressure, and total cholesterol [TC]) were developed as an evaluation system to protect against CVDs.^5^ Numerous studies have documented that ideal cardiovascular health could reduce the risk of CVD events in both subclinical^6^ and clinical^7^, ^8^ populations.

Obesity is defined as having an excessive amount of body fat. BMI is frequently used in clinical practice to evaluate the degree of obesity, which is also the same in ICVHMs.^9^ Although multiple population‐based studies have linked BMI to obesity‐related comorbidities and mortality risk,^10^ individuals with close BMI but different body fat distribution could present different metabolic status.^11^ Robust evidence showed that abdominal obesity and excess visceral adiposity highly correlated with CVD and could be more useful than BMI for detecting the risk of CVD in the general population.^12^, ^13^ However, whether weight assessment in ICVHMs needs to take adipose tissue distribution into account is unclear.

To this end, in this multicenter study, we aimed to explore whether the association of BMI or visceral fat area (VFA) with CVD risk combined with other ICVHMs would be different among Chinese T2DM patients.

## 2 METHODS

### 2.1 Study population and design

This is a cross‐sectional, observational, multicenter study. 37 486 adult patients (≥18 years) diagnosed with T2DM were consecutively enrolled from June 2017 to April 2021 in 10 sites of National Metabolic Management Centers (MMCs). After excluding participants with missing data for ICVHMs, brachial‐ankle pulse wave velocity (baPWV), and VFA (*n* = 4667) or participants <40 years old (*n* = 3771), 29 048 eligible participants were finally included in the analysis. The research was limited to patients aged 40 years or above to avoid the age‐related bias as it was well established that arterial stiffness increased with advancing age. Patients completed detailed questionnaires and underwent anthropometric measurements, laboratory examinations, and diabetes complications screening at the baseline. The study was performed according to the Declaration of Helsinki. Ethical approval was obtained from Ethical Review Committee of Ruijin Hospital, Shanghai Jiaotong University School of Medicine, and all ethics committee at other participating centers. Written informed consent was obtained from all study participants.

### 2.2 Data collection

Data were collected in 10 sites of MMCs by trained staff according to a standard protocol.^14^ Demographic characteristics, lifestyle information, family history, and medical history were collected with a standard questionnaire. Diet information was collected by questioning habitual daily intake of fresh fruits and vegetables. Physical activity was assessed by self‐reported activity intensity, frequency, and duration. Self‐reported smoking status included smoking duration, smoking intensity, and time since cessation. Blood pressure were measured while seated with an automated sphygmomanometer after ≥5 min of rest. Glycated hemoglobin (HbA1c) and lipid profile were measured in local MMCs. Lipid profiles included TC, triglycerides, high‐density lipoprotein cholesterol (HDL‐c), and low‐density lipoprotein cholesterol (LDL‐c).

### 2.3 Definition of ideal cardiovascular health metrics

ICVHMs were defined following the special reports from AHA^5^ and adjusted according to previous research.^15^ The seven components include smoking status, physical activity, healthy dietary patterns, and BMI as health behavior variables and blood glucose, blood pressure, and TC as health factor variables. Among them, the cut‐point for ideal BMI was 23 kg/m^2^, which has been proposed to be more suitable for Asian individuals.^16^ Ideal VFA was defined by a cutoff point of <100cm^2^ as the value was shown to be predictive of multiple risk of obesity‐related disorders.^17^ Ideal waist circumference and waist‐to‐hip ratio was defined as < 90 cm, <0.90 in men and < 85 cm, <0.85 in women in the current analysis.^18^, ^19^ We used fruit and vegetable intake ≥500 g/d (≈ 4.5 cups/d, with reference to the US Department of Agriculture) to assess healthy dietary intake pattern because they are the key dietary components emphasized by the AHA and have been proved to be related to reducing CVD risks.^20^ Fasting blood glucose was replaced by HbA1c as the metric to estimate glucose metabolism, owing to the recommendations from American Diabetes Association for using HbA1c as the therapeutic goals in clinical practice.^21^ Ideal HbA1c level was defined as HbA1c level <6.5%. Other metrics were described as never smoked or ceased smoking for >12 months, sufficient physical activity (≥150 mins/wk for moderate intensity or ≥ 75 mins/wk for vigorous intensity or ≥ 150 mins/wk for moderate plus vigorous intensity), TC <200 mg/dL (unmedicated, to convert the value to mmol/L), and blood pressure <120/80 mm Hg (unmedicated).

### 2.4 Measurements of VFA and baPWV

VFA and subcutaneous fat area (SFA) were measured by the dual‐impedance analysis method (DUALSCAN, HDS‐2000; Omron Healthcare Co., Ltd., Kyoto, Japan) according to the following procedure. During this process, trained staff measured the cross‐sectional area of the abdomen at the level of the umbilicus with the attached abdominal measuring unit. Electrical current was respectively applied to electrodes on hands and feet and eight electrodes placed at the level of the umbilicus. The measured impedance and abdominal shape data were used to obtain SFA and VFA. This method for measuring VFA has been shown to have excellent correlation with abdominal computed tomography.^22^

The baPWV was analyzed by a noninvasive automated device (BP‐203RPE III form PWV/ABI; Omron Healthcare Co., Ltd., Kyoto, Japan). For details of operation, refer to previously published papers.^23^ Normal arterial stiffness was defined as mean bilateral baPWV <1400 cm/s.^24^

### 2.5 Statistical analysis

Data analysis was performed using R version 4.0.5. Continuous variables are reported as mean ± SD or median (interquartile range) for normally or nonnormally distributed variables. Categorical variables are presented as percentages. The normal distribution of data was tested with the Kolmogorov–Smirnov test. For intergroup differences, categorical data were analyzed using a chi‐square test and independent samples *t* tests and nonparametric tests were used for continuous data. Multivariable associations and odds ratios (ORs) were obtained using binary logistic regression models. To better understand the pattern of associations, BMI was categorized into six clinically relevant categories using cut‐points 18.5, 23, 27.5, 32.5, and 37.5 kg/m^2^.^10^ VAT VFA was categorized into five categories using cut‐points 50, 100, 150, 200 cm^2^. We also repeated the analyses stratified by sex and age group (<60 and ≥ 60 years) and tested for interactions by including a cross‐product term in the models.

## 3 RESULTS

A total of 29 048 participants >40 years were enrolled in this study after exclusion of patients with any missing ICVHMs data, VFA data, and baPWV data at the baseline. Patient characteristics are given in Table 1. The mean (SD) age was 57.26 (8.78) years, and 16 031 were men (55.2%). The mean VFA was 99.64 (41.61) cm^2^, and the mean baPWV was 1639.60 (328.89) cm/s. Of the 29 048 patients, 21 993 (75.7%) had baPWV over 1400 cm/s and were defined as arterial stiffness in this study. Participants with higher baPWV tended to be older, with longer duration of diabetes, more frequently women, lower educated, less physically activity, had higher BMI, poorer metabolic control status, and lower number of ICVHMs. They were more likely to have a history of hypertension and hyperlipidemia. Moreover, compared to the participants with lower baPWV group, those with higher baPWV had higher level of VFA (101.41[40.37] vs. 94.12 [40.87] cm^2^) and SAT (186.27 [63.08] vs. 175.89 [67.49] cm^2^). Participants with higher baPWV also had higher waist circumferences and waist‐to‐hip ratios.

**TABLE 1:** Demographic and clinical parameters of study patients.

Baseline characteristics	Overall	baPWV<1400	baPWV≥1400	*p*
Participants	29 048	7055	21 993	
Age, years	57.26 ± 8.78	52.59 ± 7.89	58.76 ± 8.52	<.01
Male sex, *n* (%)	16 031 (55.2%)	4275 (60.6%)	11 756 (53.5%)	<.01
Education level‐High school and above, *n* (%)	10 781 (37.2%)	3064 (43.5%)	7717 (35.1%)	<.01
Drinking	3327 (11.5%)	833 (11.9%)	2494 (11.4%)	.30
Family history of diabetes	14 133 (52.1%)	3461 (52.7%)	10 672 (51.9%)	.25
Diabetic duration, year	7.69 ± 7.03	5.31 ± 5.75	8.45 ± 7.23	<.01
Smoking status				
Current	6443 (22.4%)	1853 (26.5%)	4590 (21.0%)	<.01
Former, quit≤12 mo	726 (2.5%)	227 (3.3%)	499 (2.3%)	.04
Never or quit>12 mo	21 640 (75.1%)	4910 (70.2%)	16 730 (76.7%)	<.01
BMI, kg/m^2^	25.83 ± 3.56	25.69 ± 3.68	25.87 ± 3.52	<.01
BMI levels				
<23	5848 (20.2%)	1559 (22.1%)	4289 (19.5%)	<.01
≥23	23 167 (79.8%)	5488 (77.9%)	17 678 (80.5%)	<.01
Waist circumference, cm	91.36 ± 9.65	90.45 ± 9.81	91.66 ± 9.58	<.01
Hip circumference, cm	97.22 ± 7.28	97.26 ± 7.34	97.21 ± 7.27	.59
Waist‐to‐hip ratio	0.94 ± 0.06	0.93 ± 0.06	0.94 ± 0.06	<.01
VFA, cm^2^	99.64 ± 41.61	94.12 ± 40.87	101.41 ± 40.37	<.01
SFA, cm^2^	183.75 ± 64.33	175.89 ± 67.49	186.27 ± 63.08	<.01
baPWV, cm/s	1639.60 ± 328.89	1266.39 ± 104.64	1759.32 ± 283.44	<.01
Physical activity at goal	2788 (9.7%)	863 (12.4%)	1925 (8.8%)	<.01
Fruit and vegetable intake at goal	15 790 (55.2%)	3928 (56.6%)	11 862 (54.7%)	<.01
Total cholesterol, mmol/L	4.95 ± 1.31	4.90 ± 1.28	4.97 ± 1.32	<.01
HDL cholesterol, mmol/L	1.22 ± 0.34	1.21 ± 0.34	1.22 ± 0.34	.05
LDL cholesterol, mmol/L	2.96 ± 1.00	2.94 ± 0.94	2.97 ± 1.02	.04
Systolic blood pressure, mm Hg	133.19 ± 19.04	122.13 ± 15.53	136.74 ± 18.70	<.01
Diastolic blood pressure, mm Hg	77.32 ± 11.54	74.02 ± 10.52	78.37 ± 11.65	<.01
Glycated hemoglobin A1c %	8.61 ± 2.10	8.56 ± 2.27	8.62 ± 2.04	.04
ICVHMs, no.				
≤1	6977 (24.0%)	1402 (19.9%)	5575 (25.4%)	<.01
2	9780 (33.7%)	2003 (28.4%)	7777 (35.4%)	<.01
3	7821 (26.9%)	2017 (28.6%)	5804 (26.4%)	.01
4	3334 (11.5%)	1134 (16.1%)	2200 (10.0%)	<.01
≥5	1136 (3.9%)	499 (7.0%)	637 (2.9%)	<.01
History of hypertension, *n* (%)	13 406 (46.42%)	2040 (29.08%)	11 366 (51.98%)	<.01
History of hyperlipidemia, *n* (%)	8977 (31.09%)	2021 (28.81%)	6956 (31.83%)	<.01

*Note*: Values are mean (SD) or number (percentage).

Abbreviations: baPWV, brachial‐ankle pulse wave velocity; BMI, body mass index; HDL, high density lipoprotein; ICVHM, ideal cardiovascular health metrics; LDL, low density lipoprotein; SAT, subcutaneous adipose tissue; SFA, subcutaneous fat area; VFA, visceral fat area.

Table 2 showed the ORs for baPWV dichotomous categories (≥ or < 1400 cm/second), based on the ICVHM components including different obesity parameters, respectively. After adjusting for sex, age, duration of diabetes, history of stroke, coronary heart disease (CHD), and other ICVHM components, the ORs for baPWV ≥1400 cm/s were 1.08 (95% confidence interval [CI], 1.00–1.17) for lower BMI and 0.85 (95% CI, 0.80–0.90) for lower VFA. Participants with lower waist‐to‐hip ratios also had decreased ORs for arterial stiffness (unadjusted OR, 0.64 [95% CI, 0.59–0.68]; adjusted OR, 0.86 [95% CI, 0.79–0.93]). The ratio of visceral‐to‐subcutaneous fat area or visceral‐to‐total fat area and waist circumference did not show significant association with the presence of arterial stiffness in the multivariate‐adjusted model.

**TABLE 2:** Association of the presence of arterial stiffness with different obesity parameters.

	Model 1		Model 2		Model 3	
	OR (95% CI)	*p*	OR (95% CI)	*p*	OR (95% CI)	*p*
BMI < 23 kg/m^2^	0.85 (0.80–0.91)	<.01	0.80 (0.75–0.86)	<.01	1.08 (1.00–1.17)	.05
VFA < 100 cm^2^	0.73 (0.69–0.77)	<.01	0.68 (0.64–0.72)	<.01	0.85 (0.80–0.90)	<.01
VFA/SFA	1.06 (0.93–1.22)	.38	1.19 (1.03–1.39)	.02	0.87 (0.75–1.00)	.07
VFA/TFA	1.55 (1.11–2.18)	.01	2.22 (1.53–3.21)	<.01	0.79 (0.53–1.17)	.24
WC	1.01 (1.01–1.02)	<.01	1.01 (1.01–1.02)	<.01	1.00 (0.99–1.00)	.21
Lower WC	0.75 (0.70–0.79)	<.01	0.81 (0.77–0.86)	<.01	1.03 (0.97–1.10)	.35
WHR	1.03 (1.03–1.04)	<.01	1.03 (1.03–1.04)	<.01	1.02 (1.01–1.02)	<.01
Lower WHR	0.64 (0.59–0.68)	<.01	0.69 (0.65–0.75)	<.01	0.86 (0.79–0.93)	<.01

*Note*: Model 1: Unadjusted. Model 2: Adjusted for age, sex, duration of diabetes, history of stroke and CHD. Model 3: Adjusted for age, sex, duration of diabetes, history of stroke and CHD, intensity (cigarettes smoked per day) and duration (years smoked) of smoking. Other six individual cardiovascular health metrics were also adjusted. Lower WC was defined as <90 cm in men and < 85 cm in women.^18^ Lower WHR was defined as <0.90 in men and <0.85 in women.^19^

Abbreviations: BMI, body mass index; CHD, coronary heart disease; CI, confidence interval; CVD, cardiovascular disease; OR, odds ratio; SFA, subcutaneous fat area; TFA, total fat area; VFA, visceral fat area; WC, waist circumference; WHR, waist‐to‐hip ratio.

**FIGURE 1:**
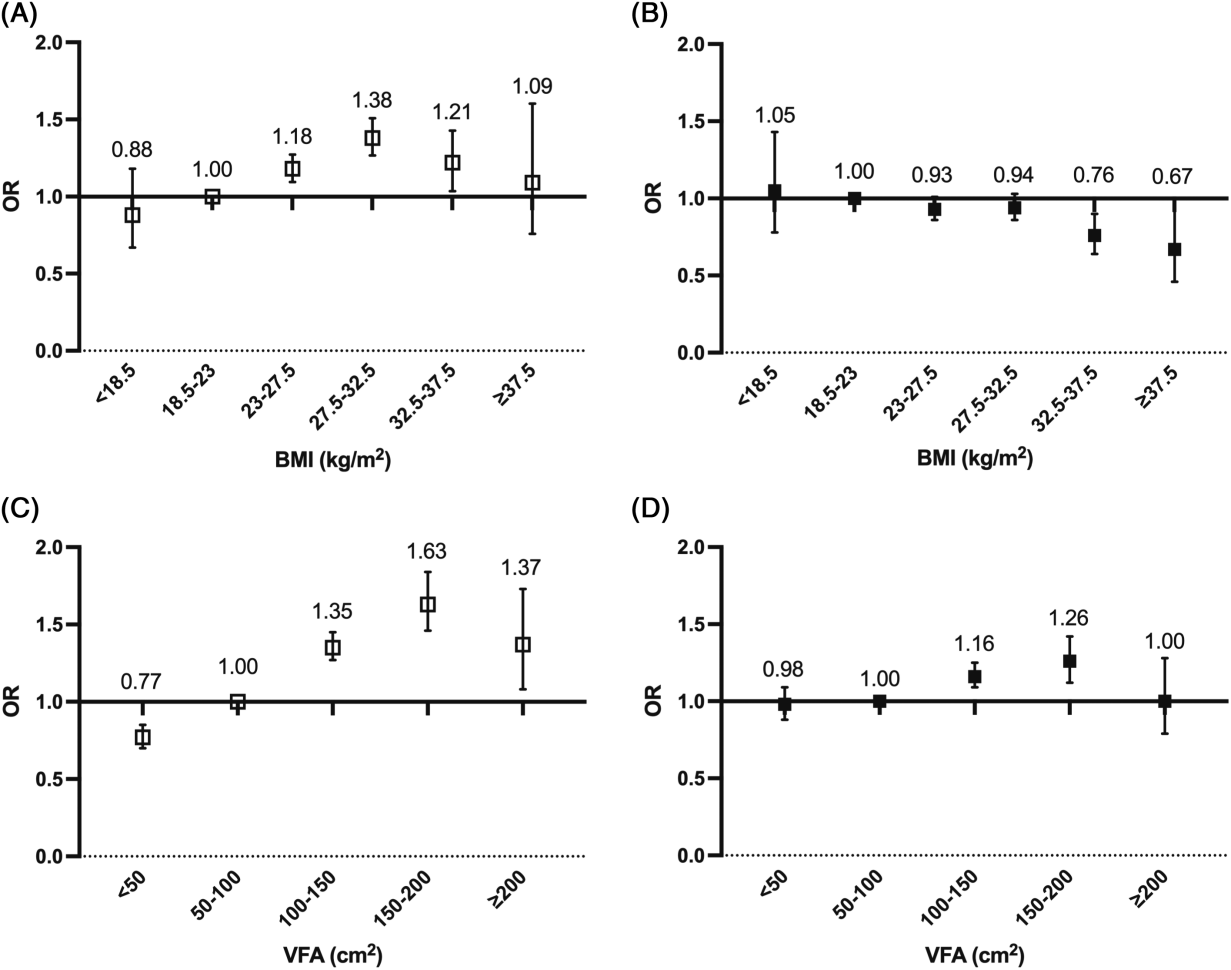
Odds ratio of arterial stiffness by BMI (A, B) and VFA (C, D) categories. Numbers above each box present odds ratios and the vertical lines represent 95% confidence intervals. White boxes are odds ratios adjusted for age, sex, duration of diabetes, and history of stroke and CHD. Black boxes are odds ratios adjusted for age, sex, duration of diabetes, history of stroke and CHD, and other ideal cardiovascular health metrics. BMI, body mass index; CHD, coronary heart disease; OR, odds ratio; VFA, visceral fat area.

**FIGURE 2:**
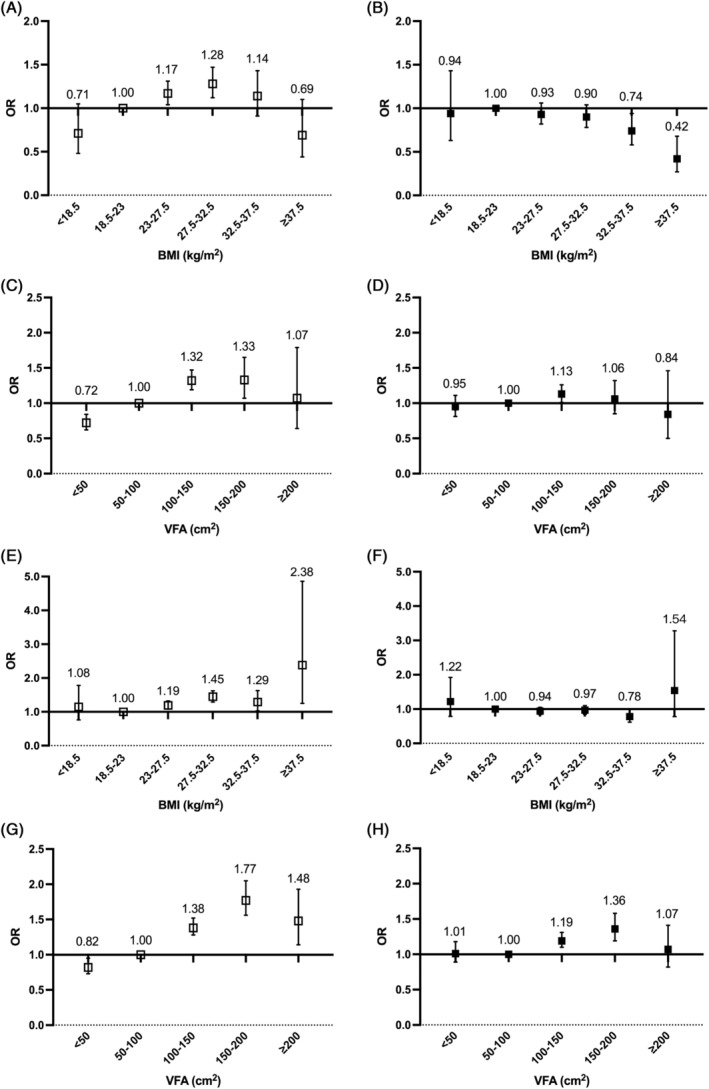
Subgroup analysis by sex of the association between the BMI, VFA, and arterial stiffness. Odds ratio of arterial stiffness by BMI categories in female (A, B) and male (E, F) patients. Odds ratio of arterial stiffness by VFA categories in female (C, D) and male (G, H) patients. Numbers above each box present odds ratios and the vertical lines represent 95% confidence intervals. White boxes are odds ratios adjusted for age, duration of diabetes, and history of stroke and CHD. Black boxes are odds ratios adjusted for age, duration of diabetes, history of stroke and CHD, and other ideal cardiovascular health metrics. BMI, body mass index; CHD, coronary heart disease; OR, odds ratio; VFA, visceral fat area.

A different association was observed between BMI and VFA with the risk of arterial stiffness in categorical analysis. Compared to participants with BMI levels in the range of 18.5–23 kg/m^2^, participants in the higher category of BMI (27.5–32.5 kg/m^2^) had a 38% higher risk of arterial stiffness after adjustment for age, sex, duration of diabetes, and history of stroke and CHD (adjusted OR, 1.38 [95% CI, 1.27–1.51]). However, after further adjustment for other ICVHM components, participants with highest BMI (>37.5 kg/m^2^) showed lowest OR of arterial stiffness (adjusted OR, 0.67 [95% CI, 0.46–1.00]). In contrast, risk of arterial stiffness rose with an increasing category of VFA. The highest risk of arterial stiffness was observed for participants with the VFA in the range of 150–200 cm^2^ before and after further adjustment (unadjusted OR, 1.63 [95% CI, 1.46–1.83]; adjusted OR, 1.26 [95% CI, 1.12–1.41]) (Figure 1). Similar trends were observed in subgroup analysis stratified by sex (Figure 2) and age (Figure S1) except that the highest risk of arterial stiffness was observed in the VAT VFA group of 100–150 cm^2^ in female and those with older age (≥60 years) in the multivariate‐adjusted model.

**FIGURE 3:**
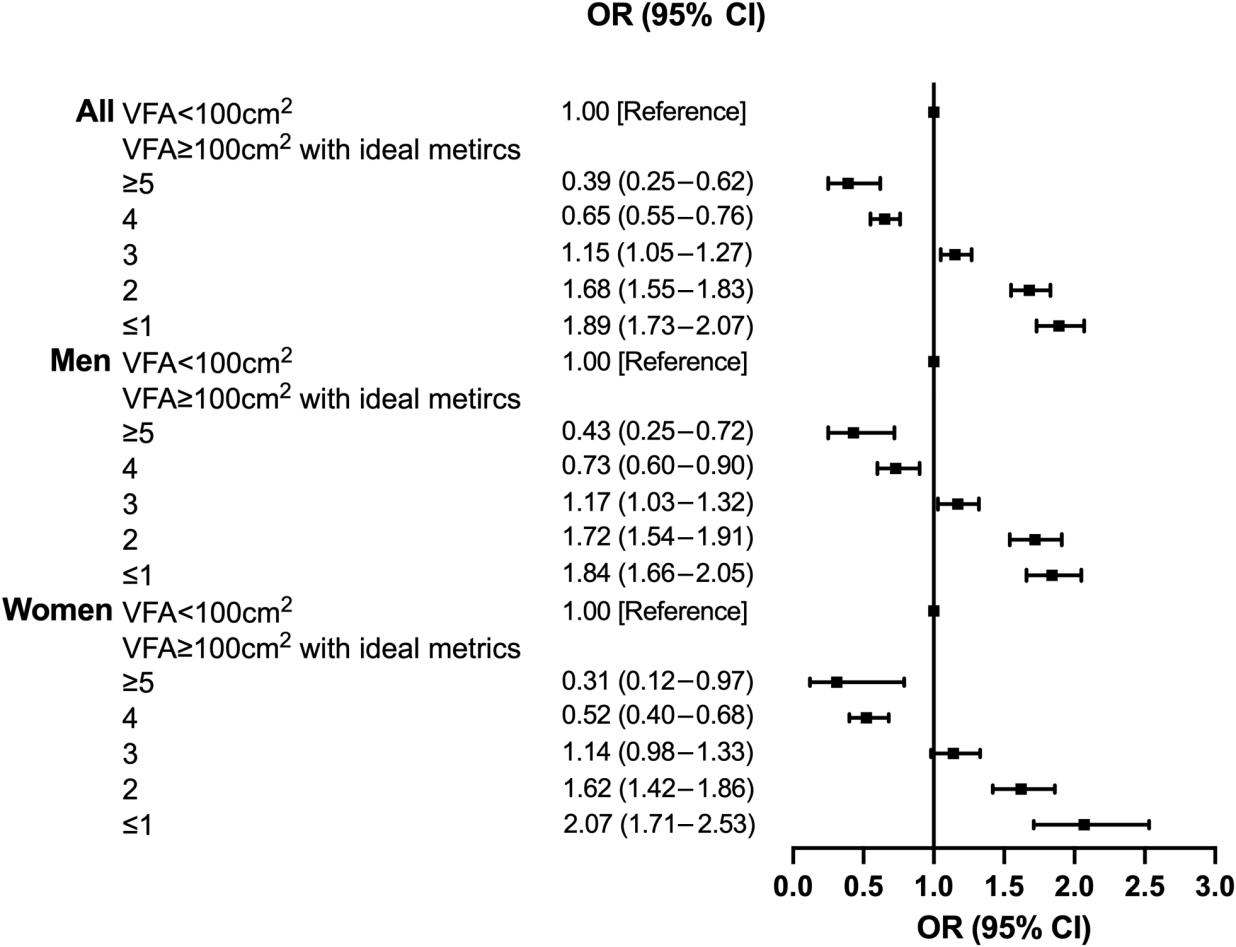
Interaction between the combination of VFA with number of ICVHMs and sex on arterial stiffness: *p* for interaction = .99. Black boxes present odds ratios and the horizontal lines represent 95% confidence intervals. Adjusted for age, duration of diabetes, and history of stroke and CHD. BMI, body mass index; CHD, coronary heart disease; ICVHMs, ideal cardiovascular health metrics; OR, odds ratio; VFA, visceral fat area.

Compared to participants with VFA < 100 cm^2^, participants with higher VFA had gradually decreased OR for arterial stiffness, from 1.89 (95% CI, 1.73–2.07) in patients who had ≤1 ICVHM to 0.39 (95% CI, 0.25–0.62) in patients who had ≥5 ICVHMs. The magnitude of the incremental risk for arterial stiffness was not associated with sex (*p* for interaction = .99) or age (*p* for interaction = .84) (Figure 3 and Figure S2). The risk for arterial stiffness also decreased with an increasing number of ICVHMs in participants with BMI ≥23 kg/m^2^ (≤1 ICVHM: OR, 1.70 [95% CI, 1.55–1.86]; ≥5 ICVHMs: OR, 0.37 [95% CI, 0.28–0.49]) (Figure S3). The magnitude of the incremental risk for arterial stiffness associated with fewer ICVHMs was greater in patients younger than 60 years (younger and ≥5 ICVHMs: OR, 0.34 [95% CI, 0.24–0.47]; ≤1 ICVHM: OR, 1.70 [95% CI, 1.54–1.89]; older and ≥5 ICVHMs: OR, 0.49 [95% CI, 0.25–0.68]; ≤1 ICVHM: OR, 1.45 [95% CI, 1.22–1.72]; *p* for interaction <.01) (Figure S4).

## 4 DISCUSSION

In this Chinese multicenter cross‐sectional study of 29 048 T2DM patients, participants with higher VFA exhibited higher risk of arterial stiffness whereas lower BMI was not an independent protective factor for lower baPWV. VFA showed better correlation when further combined with other ICVHM components. Similar results were observed in participants of different ages and of both sexes. Participants with VFA >100 cm^2^ who possessed more than three ICVHMs exhibited lower risk of arterial stiffness compared with those with normal VFA level. To our knowledge, this study is the first to comprehensively compare the predictive efficacy association of BMI and VFA with arterial stiffness combined with the other six ideal cardiovascular health metrics in patients with T2DM.

Health behaviors are strongly associated with greater longevity and CVD‐free survival. Akesson et al observed that women with healthy eating habits and lifestyle had lower risks for myocardial infarction.^25^ Similar protective effects of healthy cardiovascular behaviors also appeared in males.^26^ Numerous studies also support the prognostic importance of reasonable control of blood pressure, blood glucose, and blood lipids. Framingham Heart Study demonstrated the association of health behaviors and health factors with disease‐free survival and overall survival in the elderly.^27^ Based on these conclusions, the AHA proposed a new concept, cardiovascular health, and determined the indicators for subsequent monitoring over time.^5^

Even though obesity is considered as a major risk factor for CVDs, the relation between body weight and mortality among T2DM patients remains unresolved. Several studies have found that a higher BMI may be associated with a better survival in various chronic diseases. This paradoxical protective effect of obesity has been termed as the “obesity paradox.”^28^, ^29^ Meanwhile, although BMI is convenient to reflect the prevalence of obesity on a population level, it does not take into account the distribution of different fat depots, leading to the fact that subjects could vary considerably in cardiometabolic characteristics even with similar BMI. There is no evidence of an “obesity paradox” based on a direct body composition measurement**s**, therefore, the “obesity paradox” is not equal to the visceral adiposity paradox. The concept of central obesity was proposed by Vague et al in the 1940s^30^ and has been increasingly recognized as the core factor linked to CVD.^31^ The Framingham Heart Study suggested that visceral adipose tissue volume was associated with incident metabolic risk factors beyond overall adiposity.^32^ Liu et al have found that excess adipose tissue in the abdominal region was associated with risk factors for poor cardiometabolic health in the Jackson Heart Study.^33^ These results prompted us to consider that VFA may be more associated with CVD risk than BMI, as a better definition of obesity in the ICVHMs in T2DM patients.

In this study, visceral fat area was assessed by bioelectrical impedance analysis as it was easy, safe to use, noninvasive, with low cost and more adaptability to clinical practice.^34^ BaPWV was used as an indicator of arterial stiffness as it was considered the gold standard for the arterial stiffness measurement^35^ and linked to increased risk of cardiovascular events.^36^ Controversies remained in the relationship between BMI and baPWV.^37^, ^38^ BaPWV showed a significant positive correlation with BMI alone but was significantly lower as BMI increased when adjusting for age, gender, and other cardiovascular risk factors.^39^ Interestingly, we detected that the positive correlation between BMI and baPWV disappeared when other ICVHMs were adjusted. Among T2DM individuals, patients with VFA ≥100cm^2^ showed higher risk of baPWV >1400 cm/s, whereas a similar association was not found in individuals with BMI ≥23 kg/m^2^. The group with VFA in the range of 150–200 cm^2^ showed highest odds ratio of arterial stiffness. Meanwhile, risk of arterial stiffness also decreased in patients with lower waist‐to‐hip ratio, which further illustrated that central obesity was more important in evaluating cardiovascular health status in T2DM patients. With the advantage of precise measurement and clearly defined cut‐point, we believe that VFA can be more suitable for quantifying weight control in the concept of ideal cardiovascular health in T2DM patients. Based on this finding, we suggest that  VFA may be a better alternative when assessing obesity in ICVHMs in patients with T2DM if conditionally possible. We also detected that patients with VFA ≥100cm^2^ who had four or more ICVHMs showed lower risk of arterial stiffness than patients with lower VFA. We speculated that the more ICVHMs were developed, the more adverse effect of higher VFA might be counteracted, which again highlighting the importance of maintaining other health behaviors and health factors.

Sexual dimorphism in the distribution of underlying fat composition was also well established. Adipose tissue is more likely to accumulate in the trunk and abdomen in males than in females,^40^ which was in keeping with our results. The fact that women have a predominance of subcutaneous fat might imply a lower relative risk of CVD for women than men with a similar BMI. Furthermore, Britton et al reported that VFA was a stronger harbinger of incident CVD in men from the Framingham Heart Study cohort.^41^ In parallel, Kammerlander et al indicated that VFA conferred a greater association with incident cardiometabolic risk factors and CVD events in women, when compared with BMI. However, same outcome has not been obtained in men.^42^ In our study, it seems that VFA were associated with a markedly greater risk for higher baPWV level compared with BMI, particularly in men. Male patients with a VFA level from 150 to 200 cm^2^ had highest risk of arterial stiffness (adjusted OR, 1.36 [95% CI 1.18–1.56]). In women, the highest risk of arterial stiffness was observed in the VFA group of 100–150 cm^2^ (adjusted OR, 1.13 [95% CI 1.01–1.26]). Considering the sex‐related differences in adiposity distribution and its impact on arterial stiffness, identical VFA cutoff values for men and women would be inappropriate. Sex‐specific thresholds for VFA need to be determined in future.

The present analysis had several limitations that need to be addressed. First, our study was restricted to T2DM patients, which may limit the ability to generalize these findings to other populations. Second, in the analyses reported here, we focused on several indicators for central obesity. We did not measure the total fat content limited to the original study design. Finally, with the cross‐sectional design, this study could not establish cause–effect relationships. As we included only baseline cardiovascular health status, it was unable to quantify the effects of changes in the ICVHM profile on arterial stiffness. The prospective long‐term follow‐up data from this cohort might verify the findings from this study. However, our study had a number of strengths including the large sample size, standardized assessment of VFA, and complete collection of seven components of ideal cardiovascular health metrics. In the meantime, as far as we know, this study proposed for the first time compare the difference of VFA and BMI in goal‐setting to promote overall cardiovascular health status.

In conclusion, our study revealed that there was a significant association between VFA and higher baPWV. The combination of VFA and other ideal cardiovascular health metrics had good prediction effect on arterial stiffness. VFA might be a better measurement of body fatness than BMI in evaluating cardiovascular health status in T2DM patients. Our findings indicated a potential advantage of using visceral adiposity, for evaluating obesity instead of BMI ICVHMs in patients with T2DM.

## CONFLICT OF INTEREST STATEMENT

The authors report no conflicts of interest in this work.

## Supporting information


**Figure S1.** Subgroup analysis by age of the association between the BMI, VFA and arterial stiffness in young (40‐ < 60 years) (A‐D) and older aged (≥60 years) (E‐H) groups. Numbers above each box present odds ratios and the vertical lines represent 95% confidence intervals. White boxes are odds ratios adjusted for sex, duration of diabetes and history of stroke and CVD. Black boxes are odds ratios adjusted for sex, duration of diabetes, history of stroke and CVD and other ideal cardiovascular health metrics. BMI, body mass index; CVD, cardiovascular disease; OR, odds ratio; VFA, visceral fat area.

**Figure S2.** Interaction between the combination of VFA with number of ICVHMs and age on arterial stiffness: P for interaction = 0.84. Black boxes present odds ratios and the horizontal lines represent 95% confidence intervals. Adjusted for sex, duration of diabetes, and history of stroke and CVD. CI, confidence interval; CVD, cardiovascular disease; ICVHMs, ideal cardiovascular health metrics; OR, odds ratio; VFA, visceral fat area;

**Figure S3.** Interaction between the combination of BMI with number of ICVHMs and sex on arterial stiffness: *p* for interaction = .56. Black boxes present odds ratios and the horizontal lines represent 95% confidence intervals. Adjusted for age, duration of diabetes, and history of stroke and CVD. BMI, body mass index; CI, confidence interval; CVD, cardiovascular disease; ICVHMs, ideal cardiovascular health metrics; OR, odds ratio.

**Figure S4.** Interaction between the combination of BMI with number of ICVHMs and age on arterial stiffness: P for interaction <0.01. Black boxes present odds ratios and the horizontal lines represent 95% confidence intervals. Adjusted for sex, duration of diabetes, and history of stroke and CVD. BMI, body mass index; CI, confidence interval; CVD, cardiovascular disease; ICVHMs, ideal cardiovascular health metrics; OR, odds ratio.
