# Supplementary figures and images for: Association of visceral fat area or BMI with arterial stiffness in ideal cardiovascular health metrics among T2DM patients
Source: J Diabetes. 2023 Sep 8;16(1):e13463. doi: 10.1111/1753-0407.13463 (PMC10809303; doi:10.1111/1753-0407.13463)

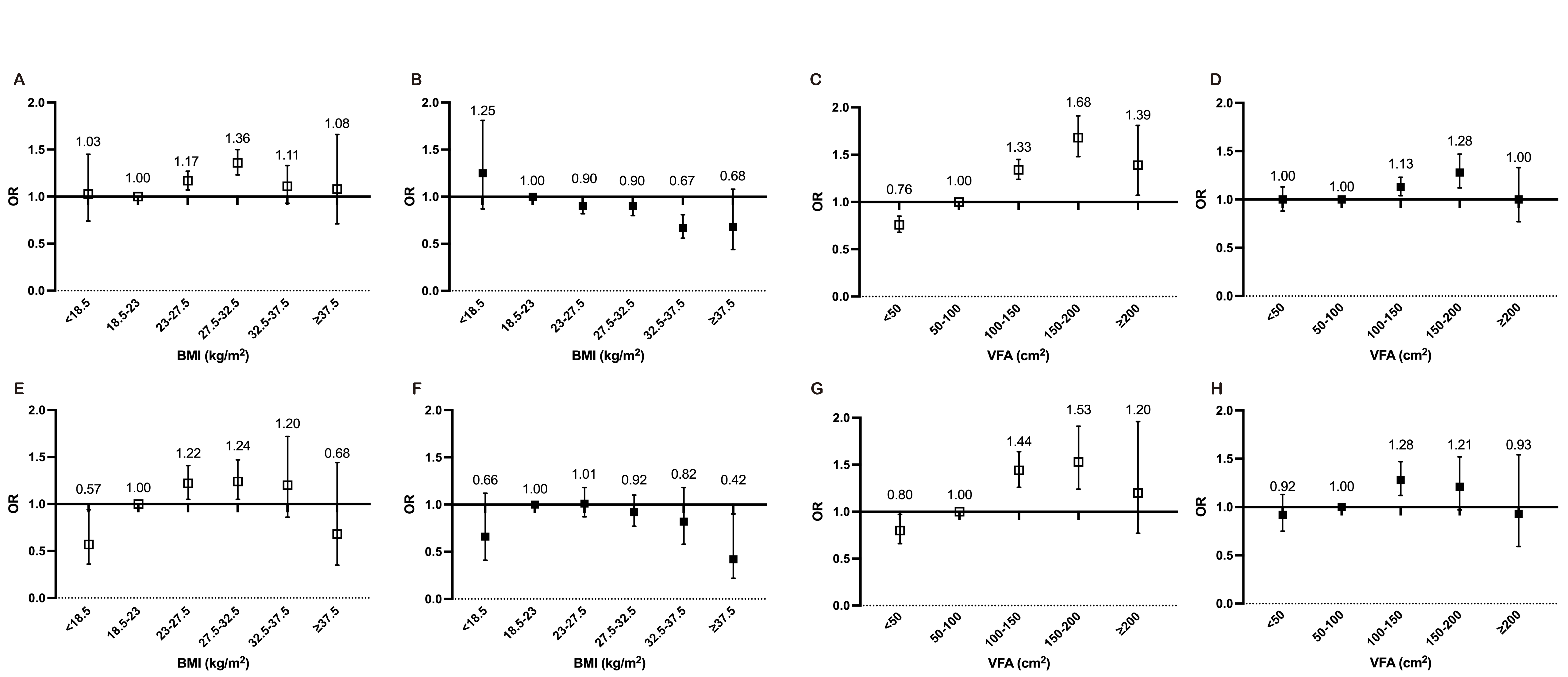

Supplement: Supplementary file 1 — Figure S1. Subgroup analysis by age of the association between the BMI, VFA and arterial stiffness in young (40‐ < 60 years) (A‐D) and older aged (≥60 years) (E‐H) groups. Numbers above each box present odds ratios and the vertical lines represent 95% confidence intervals. White boxes are odds ratios adjusted for sex, duration of diabetes and history of stroke and CVD. Black boxes are odds ratios adjusted for sex, duration of diabetes, history of stroke and CVD and other ideal cardiovascular health metrics. BMI, body mass index; CVD, cardiovascular disease; OR, odds ratio; VFA, visceral fat area. [file JDB-16-e13463-s004.tif]

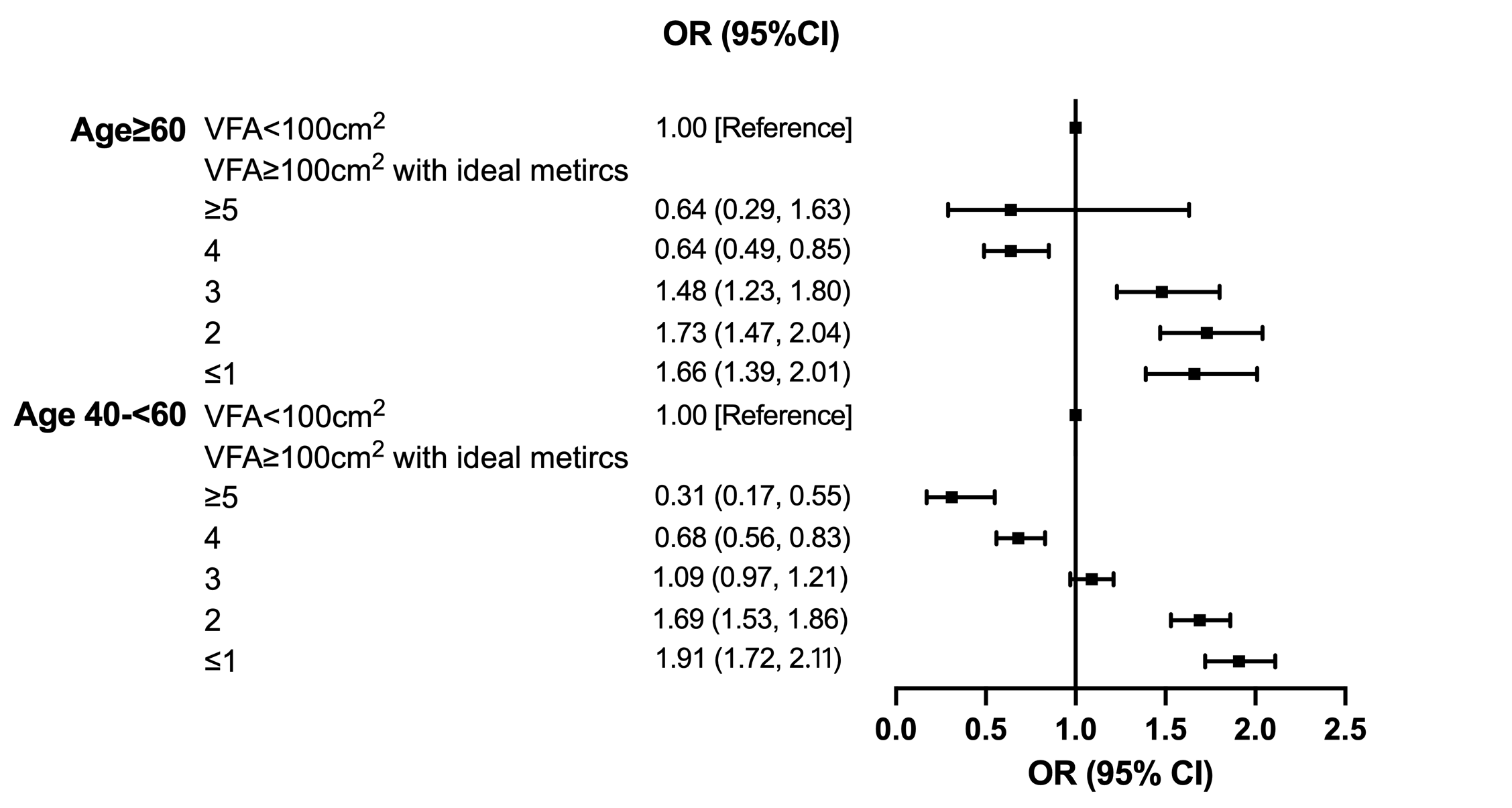

Supplement: Supplementary file 2 — Figure S2. Interaction between the combination of VFA with number of ICVHMs and age on arterial stiffness: P for interaction = 0.84. Black boxes present odds ratios and the horizontal lines represent 95% confidence intervals. Adjusted for sex, duration of diabetes, and history of stroke and CVD. CI, confidence interval; CVD, cardiovascular disease; ICVHMs, ideal cardiovascular health metrics; OR, odds ratio; VFA, visceral fat area; [file JDB-16-e13463-s001.tiff]

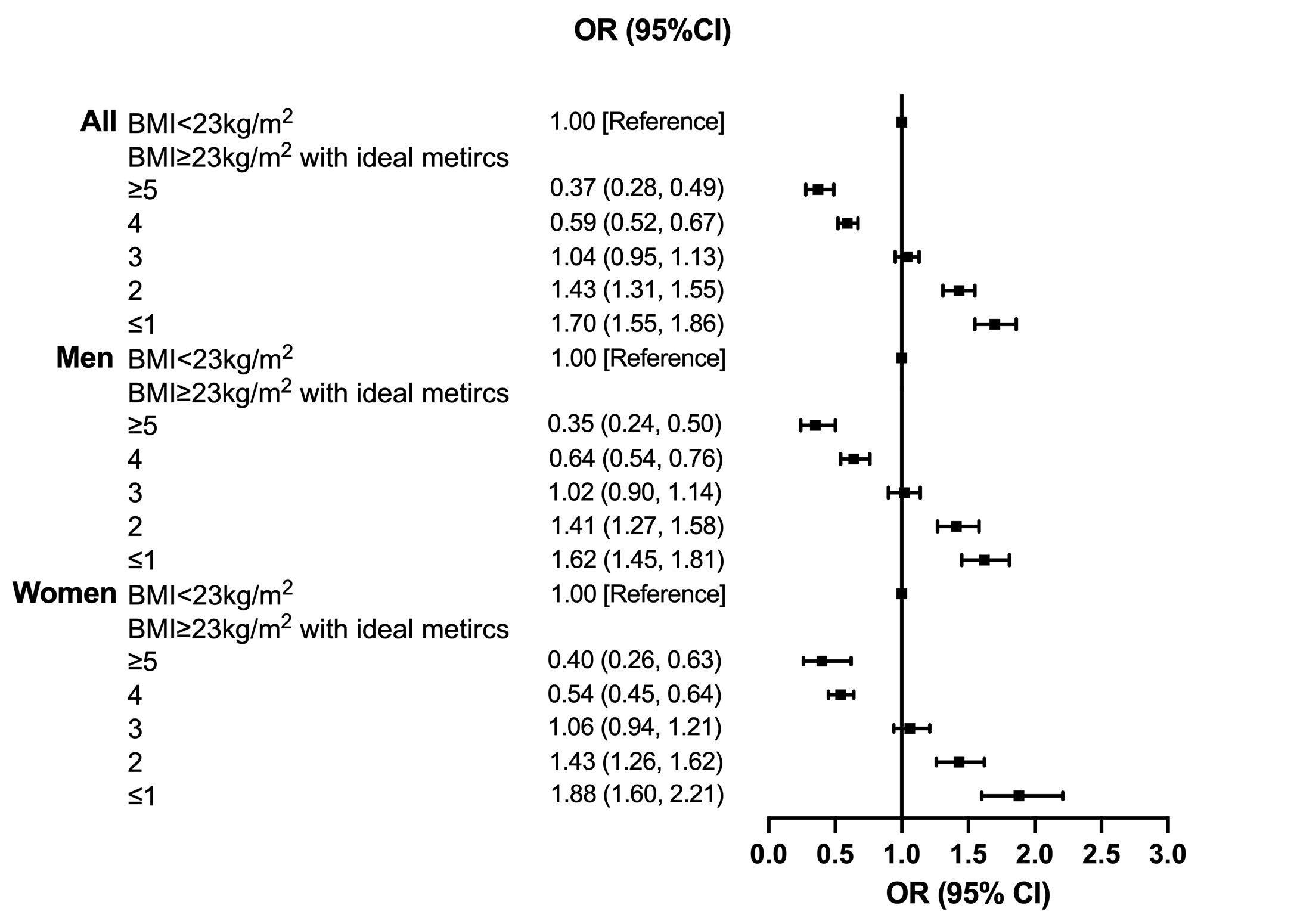

Supplement: Supplementary file 3 — Figure S3. Interaction between the combination of BMI with number of ICVHMs and sex on arterial stiffness: p for interaction = .56. Black boxes present odds ratios and the horizontal lines represent 95% confidence intervals. Adjusted for age, duration of diabetes, and history of stroke and CVD. BMI, body mass index; CI, confidence interval; CVD, cardiovascular disease; ICVHMs, ideal cardiovascular health metrics; OR, odds ratio. [file JDB-16-e13463-s002.tiff]

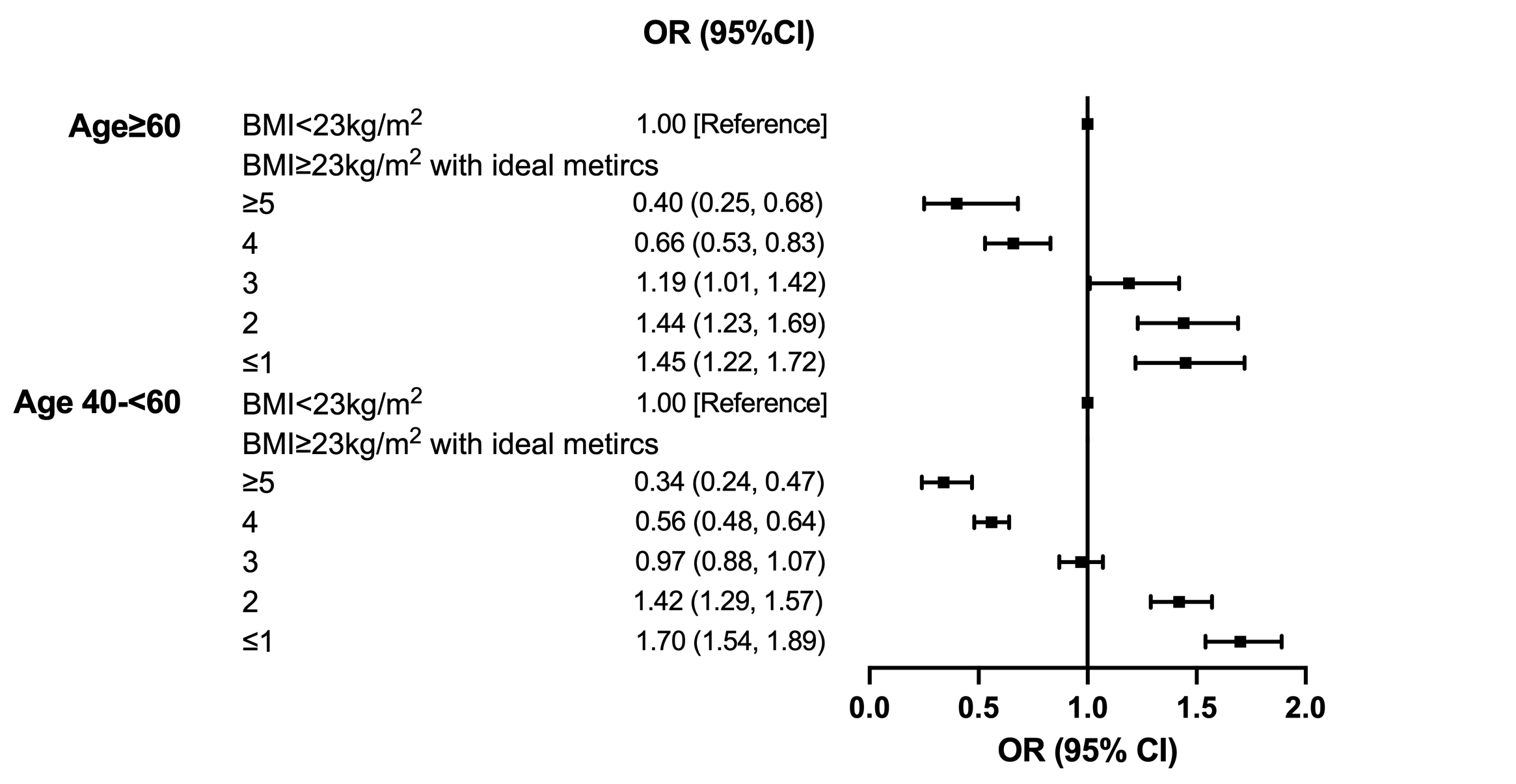

Supplement: Supplementary file 4 — Figure S4. Interaction between the combination of BMI with number of ICVHMs and age on arterial stiffness: P for interaction <0.01. Black boxes present odds ratios and the horizontal lines represent 95% confidence intervals. Adjusted for sex, duration of diabetes, and history of stroke and CVD. BMI, body mass index; CI, confidence interval; CVD, cardiovascular disease; ICVHMs, ideal cardiovascular health metrics; OR, odds ratio. [file JDB-16-e13463-s003.tiff]
